# Sputum analysis by flow cytometry; an effective platform to analyze the lung environment

**DOI:** 10.1371/journal.pone.0272069

**Published:** 2022-08-17

**Authors:** Lydia H. Bederka, Jamila R. Sanchez, Jennifer Rebeles, Patricia R. Araujo, Marcia H. Grayson, Shao-Chiang Lai, Louis R. DePalo, Sheila A. Habib, David G. Hill, Kathleen Lopez, Lara Patriquin, Robert Sussman, James Humphreys, Xavier T. Reveles, Vivienne I. Rebel

**Affiliations:** 1 bioAffinity Technologies, San Antonio, Texas, United States of America; 2 Department of Medicine, Icahn School of Medicine at Mount Sinai, New York, New York, United States of America; 3 South Texas Veterans Health Care System (STVHCS), Audie L. Murphy Memorial Veterans Hospital, San Antonio, Texas, United States of America; 4 Waterbury Pulmonary Associates LLC, Waterbury, Connecticut, United States of America; 5 Radiology Associates of Albuquerque, Albuquerque, New Mexico, United States of America; 6 Atlantic Respiratory Institute, Summit, New Jersey, United States of America; 7 Precision Pathology Services, San Antonio, Texas, United States of America; 8 Department of Cell Systems & Anatomy, The University of Texas Health Science Center at San Antonio, San Antonio, TX, United States of America; Inha University Hospital, REPUBLIC OF KOREA

## Abstract

Low dose computed tomography (LDCT) is the standard of care for lung cancer screening in the United States (US). LDCT has a sensitivity of 93.8% but its specificity of 73.4% leads to potentially harmful follow-up procedures in patients without lung cancer. Thus, there is a need for additional assays with high accuracy that can be used as an adjunct to LDCT to diagnose lung cancer. Sputum is a biological fluid that can be obtained non-invasively and can be dissociated to release its cellular contents, providing a snapshot of the lung environment. We obtained sputum from current and former smokers with a 30+ pack-year smoking history and who were either confirmed to have lung cancer or at high risk of developing the disease. Dissociated sputum cells were counted, viability determined, and labeled with a panel of markers to separate leukocytes from non-leukocytes. After excluding debris and dead cells, including squamous epithelial cells, we identified reproducible population signatures and confirmed the samples’ lung origin. In addition to leukocyte and epithelial-specific fluorescent antibodies, we used the highly fluorescent meso-tetra(4-carboxyphenyl) porphyrin (TCPP), known to preferentially stain cancer (associated) cells. We looked for differences in cell characteristics, population size and fluorescence intensity that could be useful in distinguishing cancer samples from high-risk samples. We present our data demonstrating the feasibility of a flow cytometry platform to analyze sputum in a high-throughput and standardized matter for the diagnosis of lung cancer.

## Introduction

Lung cancer accounted for an estimated 1.8 million deaths worldwide in 2020 [1]. An estimated 130,180 people will die in 2022 from lung cancer in the US alone [2]. Overall five-year survival of lung cancer remains low at 22.9% [2] because most patients present with advanced disease.

The American National Lung Screening Trial (NLST) demonstrated that LDCT screening detects 93.8% of lung cancers among high-risk individuals (i.e., people aged 55–74 with > 30 pack-years of smoking and who are currently smoking or have quit smoking in the past 15 years) [3]. The NLST showed that LDCT screening leads to an overall 20% reduction in lung cancer-specific mortality compared to screening by chest radiography [4, 5]. Unfortunately, 96.4% of positive LDCT scans were false positives in this trial, leading to approximately 90% of patients with a positive LDCT undergoing additional procedures to determine if nodules observed on their LDCT scan were cancerous [4]. These procedures, including imaging, biopsies, and surgical resection can cause serious adverse effects, including death [6].

New guidelines for interpreting LDCT scans and models to estimate the probability that a nodule is cancerous [7] improved the false positive rate (FPR) [8, 9]. Still, only a fraction of eligible patients undergoes LDCT screening [10]. A failure to communicate screening benefits and potential harms (whether due to lack of knowledge or time), expenses related to LDCT, lack of LDCT access, and repeated radiation exposure from serial LDCT scans may all contribute to low adoption of screening [11–13]. A simple, non-invasive, radiation-free, and cost-effective test that assists physicians in making, or excluding, a lung cancer diagnosis with greater certainty may decrease unnecessary follow-up procedures and increase lung cancer screening.

Sputum is an easily obtained bodily fluid that has long been part of lung cancer diagnostics. Developed by Papanicolaou and [14] optimized by Saccomanno [15, 16], the PAP sputum cytology test was the first lung cancer diagnostic, dating to the 1960s. For this test, two sputum smear slides are labeled with a PAP stain and read by a pathologist specialized in lung cytology. The sensitivity of sputum cytology is highly variable; however, its specificity is very high. A review of 16 published studies on sputum cytology including more than 28,000 patients reported a range of 42% to 97% sensitivity, with an average sensitivity of 66%, while specificity showed an average of 99% [17].

Sputum cytology’s poor sensitivity is attributed in part to inadequate samples and analysis of only a small portion of the sample [18, 19]. Inadequacy can occur because the sample produced is saliva or the mucus/debris/red blood cells within the smear obscure the cellular components needed for accurate analysis. Over time, changes to the original sputum cytology test improved its sensitivity. Nebulizers [20–22] and assist devices such as the acapella and the lung flute [18, 23], as well as patient’s adherence to proper instructions on how to produce lung sputum samples [24, 25], have shown to improve a patient’s ability to produce sputum. Liquid cytology tests [26] and automated slide preparation devices [27] can diminish the background contaminants of sputum smears and thus increase the quality of slides. Increasing the number of samples read [28–35] has also been shown to increase the likelihood of finding abnormal cells indicative of lung cancer.

Porphyrins, such as TCPP, have been known for decades to concentrate in cancers [36–38] and are currently used as diagnostic reagents in bladder cancer [39] and surgery to identify the edges of cancerous tissue [40, 41]. Using microscopy, we showed that by labeling sputum cells with the fluorescent porphyrin TCPP, we could distinguish study participants with lung cancer from those without the disease [18] with high accuracy. However, the slide-based assay is time consuming, often prohibiting the analysis of the entire sample and thereby potentially missing important events [18].

Using a flow cytometric platform, we demonstrate the feasibility of analyzing entire sputum samples without clogging the instrument. We were able to eliminate contaminants, both debris and squamous epithelial cells (SECs, common contaminants from the oral cavity [42]), using a gating strategy defined by bead standards and a viability dye. We included a quality control parameter to detect alveolar macrophages, verifying the lung origin of each sputum sample, and we defined a numerical cutoff for sample adequacy for providing reliable analysis. Lastly, staining sputum-derived cells with a cocktail of leukocyte- and epithelial-specific antibodies and TCPP allowed us to identify significant differences between samples obtained from people diagnosed with lung cancer and those obtained from people without the disease.

## Materials and methods

### Human sputum samples

A minimal risk study was registered with ClinicalTrials.gov (NCT03457415), approved by Sterling Institutional Review Board (Atlanta, GA), and conducted according to ethical principles of the Declaration of Helsinki (v 1996) and Good Clinical Practice guidelines.

Sample collection was performed at five study centers including Atlantic Health System, NJ; Mt. Sinai Hospital, NY; Radiology Associates of Albuquerque, NM; South Texas Veterans Healthcare System, TX; and Waterbury Pulmonary Associates, CT. All participants provided written consent after the study was explained to them by a study coordinator, before or after receiving a LDCT. Participants at high risk for developing lung cancer were 55 to 74 years of age, had smoked at least 30-pack years and had not quit smoking within the past 15 years. The exception was one participant who had a 45-pack year smoking history but had quit more than 15 years ago. The LDCT scans of most high-risk participants were considered not suspicious for lung cancer at the time of enrollment and participants were advised to return for LDCT screening in 12 months. A few received a recommendation for a follow-up LDCT within 3 or 6 months and those participants were followed until we received a conclusive answer regarding their health status; 3 high-risk participants were diagnosed with lung cancer and were then added to the cancer group. For the lung cancer group there were no age and smoking limitations. The age range for the cancer group was 54 to 79 years. They had smoked 6.2 to 117.5 pack-years and one participant in the cancer group was a never-smoker. Cancer was confirmed by biopsy after sputum collection. Exclusion criteria included the presence of severe obstructive lung disease, uncontrolled asthma, angina with minimal exertion and pregnancy.

**Fig 1:**
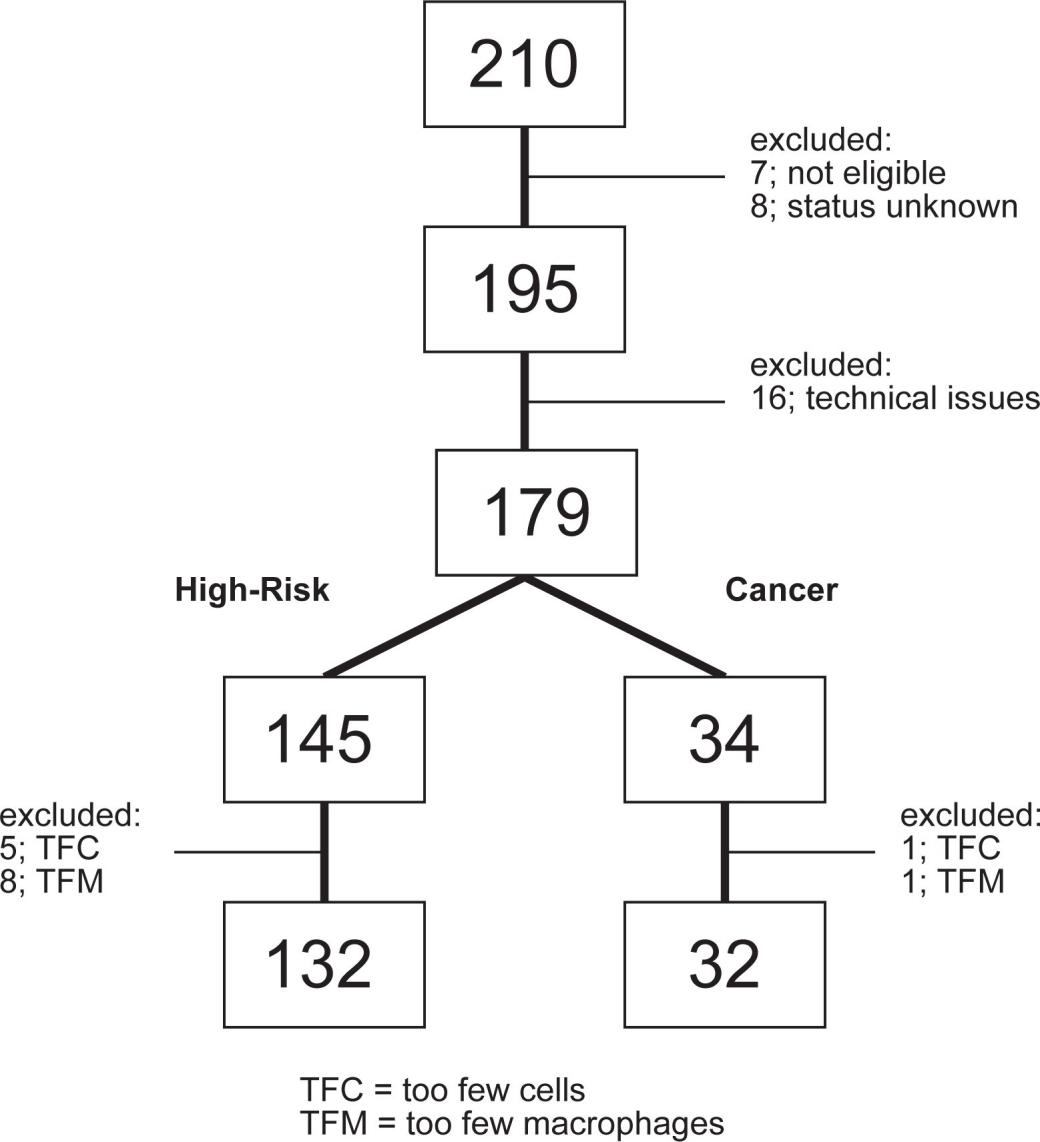
Scheme of patient sample utilization. Two hundred and ten sputum samples were collected. Thirty-one samples were initially excluded, leaving 179 samples for flow cytometric analysis. An additional 15 samples were omitted from the final analysis because of either too few cells in general (which does not amount to a reliable profile) or too few macrophages (which casts doubt to the lung origin of the sample). Ultimately 132 high-risk and 32 cancer samples were fully analyzed.

An initial 210 sputum samples were collected and analyzed by flow cytometry. Seven samples fell outside the eligibility criteria, while for eight samples the cancer/high-risk status could not be confirmed and sixteen samples were excluded because of technical issues. Upon defining the quality control parameters for sputum adequacy six more samples were excluded because of too few cells and an additional nine samples because of too few macrophages (Fig 1). Ultimately, 164 samples, including 132 high-risk and 32 cancer participants were used. Fourteen of the cancer patients were either in Stage I or II, another 14 were either Stage III or IV, and the remaining 4 had no staging information. Fifteen of the cancer patients were diagnosed with squamous cell carcinoma, thirteen with adenocarcinoma, two with small cell carcinoma and one each with large cell carcinoma and non-small cell carcinoma.

### Sample collection

All study participants were trained in using the acapella assist device (Smiths Medical, St. Paul, MN), in accordance with manufacturer’s instructions. Subjects collected sputum at home for three consecutive days, while storing their specimen cup (without preservatives) in a refrigerator. After completing the three-day collection, samples were shipped overnight to bioAffinity’s laboratory with a frozen cold pack, or in the case of South Texas Veterans Healthcare, samples were messengered to the lab with a frozen cold pack and stored in a refrigerator for processing the next day. Study researchers were blinded to the participant sample’s site of origin and all personal information of study participants.

### Sputum dissociation

Sputum was liquefied using pre-warmed 0.1% dithiothreitol (DTT) at a 1:4 ratio with sputum weight (w/v) and pre-warmed 0.5% N-acetyl-L-cysteine (NAC) at a ratio of 1:1 (w/v), as described previously [43]. The resulting cell suspension was filtered through 100 μm nylon cell strainers (Falcon, Corning Inc.) to eliminate larger debris while minimizing cell loss (S1 Fig). Cells were collected into 50 mL conical tubes, washed and centrifuged at 800 x *g* for 10 minutes. The dissociated sputum pellets were combined into one 15 mL conical tube per sputum sample. The total cell yield and viability were determined with a Neubauer hemocytometer using the trypan blue exclusion method.

### Sputum labeling for flow cytometry

Cell labeling was performed as described previously [43]. Briefly, the majority of cells were split into two tubes: one including markers to interrogate the leukocyte (CD45^+^) cell compartment and one for the epithelial (CD45^-^) cell compartment. Each tube contained the anti-CD45 antibody, FVS510 (to exclude dead cells, including SECs [44]), as well as the porphyrin TCPP (to identify cancer (associated) cells [18]). For identifying leukocyte populations, an anti-CD206 antibody was added to label macrophages as well as a cocktail of antibodies to label granulocytes (anti-CD66b) and lymphocytes (anti-CD3 and anti-CD19). For epithelial cell recognition, we used anti-cytokeratin (panCK) and anti-EpCAM. No permeabilization step was performed for the cytokeratin labeling as the initial DTT and NAC treatment for sputum processing was sufficient for intracellular cytokeratin staining [45, 46].

Dissociated sputum cells were incubated with the antibodies and FVS510 for 35 minutes. After one wash with cold HBSS, cells were fixed on ice for one hour with paraformaldehyde, after which cells were washed once again and stored on ice until TCPP labeling the next day. TCPP was added to the cells for one hour. After incubation, cells were washed twice with cold HBSS and then stored on ice until flow cytometric analysis. Throughout the labeling procedure until analysis, cells were kept on ice and protected from light. For more details about the reagents, see S1 Table.

### Flow cytometric analysis and cell sorting

Data acquisition was performed using the equipment at the University of Texas Health San Antonio Flow Cytometry Shared Resource Facility. The BD LSR II flow cytometer (BD Biosciences), equipped with 4 lasers (405nm, 488nm, 561nm and 633nm) was used to analyze samples while cell sorting was performed on a BD FACSAria cell sorter (BD Biosciences) equipped with a 100 μm nozzle and with the same 4 lasers as the LSR II. CS&T beads (BD Bioscience) were used per the manufacturer’s protocol prior to each instrument session, as passing performance tests were an additional quality control for monitoring cytometer performance. Post-collection data analysis was performed with FlowJo software (Tree Star, Inc. Ashland, OR). Cytocentrifuge slides (cytospins) of sorted cell populations were prepared and stained using the Wright-Giemsa method. Cytospins were analyzed using an Olympus microscope and images were captured and cell sizes were quantified using cellSens software (Olympus Life Science, Waltham, MA, USA). All cell types captured on cytospins were confirmed by a pathologist. Statistical analysis was performed using GraphPad software (San Diego, CA).

## Results

### Eliminating SECs from sputum sample analysis

**Fig 2:**
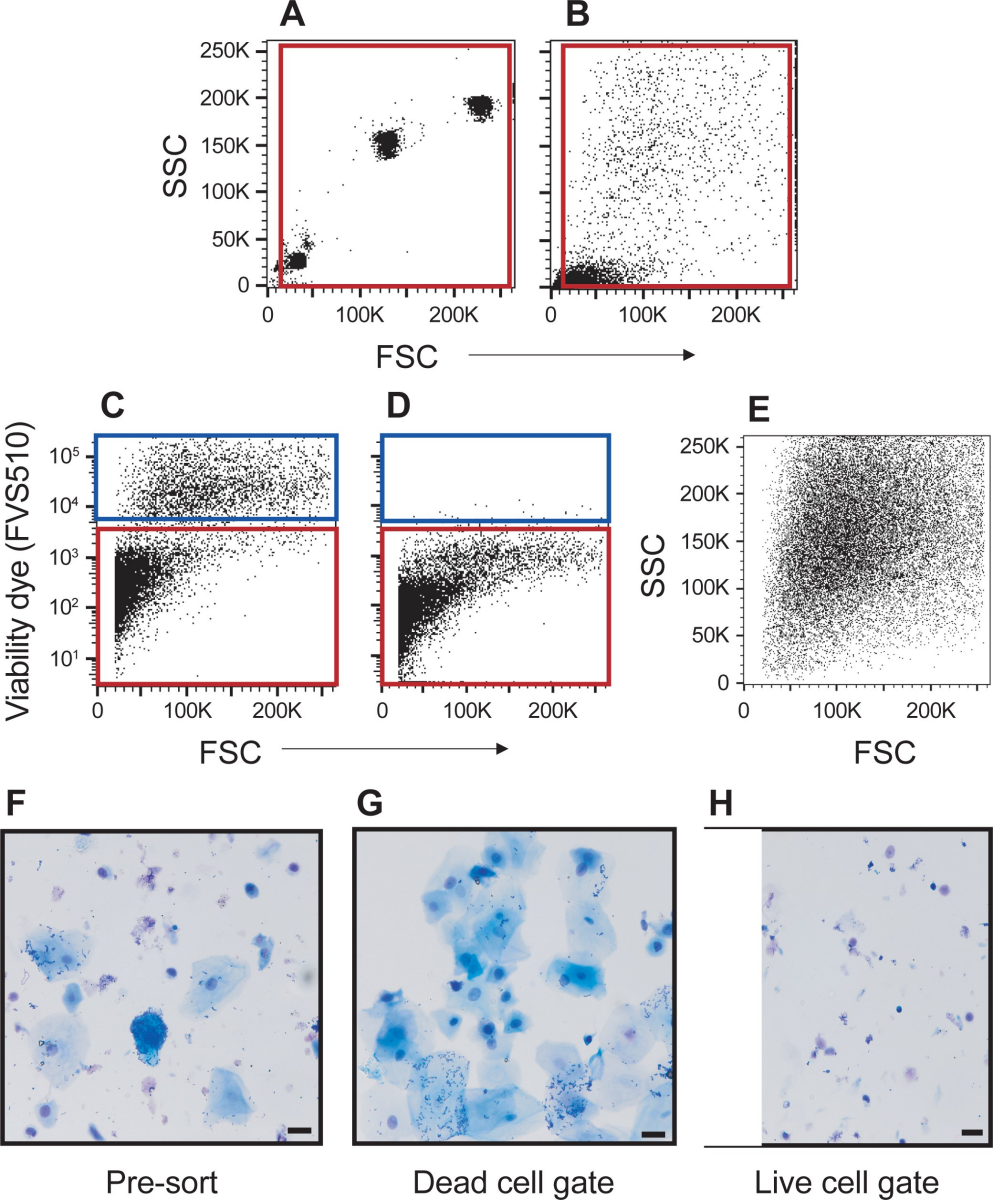
Viability dye labels squamous epithelial cells as dead cells. Light scatter profiles of 5 μm, 20 μm, and 30 μm particles (A) and dissociated sputum cells (**B**), both using the same voltage settings. The red boxes in **A** and **B** indicate a gate to exclude small debris (the bottom, left corner) as well as cell aggregates (last SSC and FSC channels). **C**) Sputum cells labeled with the FVS510 viability dye. **D**) Unstained sputum cells. The red (live cells) and blue boxes (dead cells) in **C** and **D** indicate populations of interest of sorting. **E**) Back-gating of dead cells to visualize the scatter profile of these cells. **F, G**) Wright-Giemsa-stained cells on a cytospin. The scale bars indicate 20 μm. **F**) Dissociated sputum cells prior to cell sorting. **G**) Cells collected from the dead cell gate comprised mostly of squamous epithelial cells. **H**) Cells collected from the live cell gate showing a heterogenous mixture of leukocytes and non-leukocytes.

SECs are highly auto-fluorescent and can potentially result in false positive events when sputum is analyzed by flow cytometry [47]. Therefore, elimination of SECs from the sputum sample is essential. We found that physical elimination by filtration did not work (S1 Fig), nor did a negative size selection at the time of analysis result in excluding these cells (S2 Fig). Kasai *et al*. [44] had shown that SECs in saliva are dead. We therefore tested if we could use a live/dead cell discriminator (FVS510) to eliminate SECs from the sputum analysis. Dissociated sputum cells within the 5-to-30 μm size parameters (Fig 2A and 2B; red box) were analyzed for viability (Fig 2C and 2D) as sputum cells of interest and SECs fall in to this gated area (S2 Table; S2 Fig) [47–51]. The cutoff for FVS510 positivity was based on the unstained control (Fig 2D). Back-gating the dead cells (Fig 2C; blue box) onto the sputum light scatter profile showed these cells had a general high SSC, which can be expected for SECs (Fig 2E, compare to S2Biv Fig). To confirm that SECs were dead, sputum cells were sorted into dead and live cell populations. Aliquots of the pre-sorted sample (Fig 2F) and the sorted populations were transferred to cytospins and stained with Wright-Giemsa. These slides showed that SECs are predominantly among the dead cells (Fig 2G) while live cells (Fig 2H) sorted from the same sample included hematopoietic and non-hematopoietic cells, and a few contaminating SECs. In conclusion, sputum samples can be analyzed by flow cytometry while excluding contaminating SECs with a viability gate.

### Macrophage enumeration as a quality control measurement

**Fig 3:**
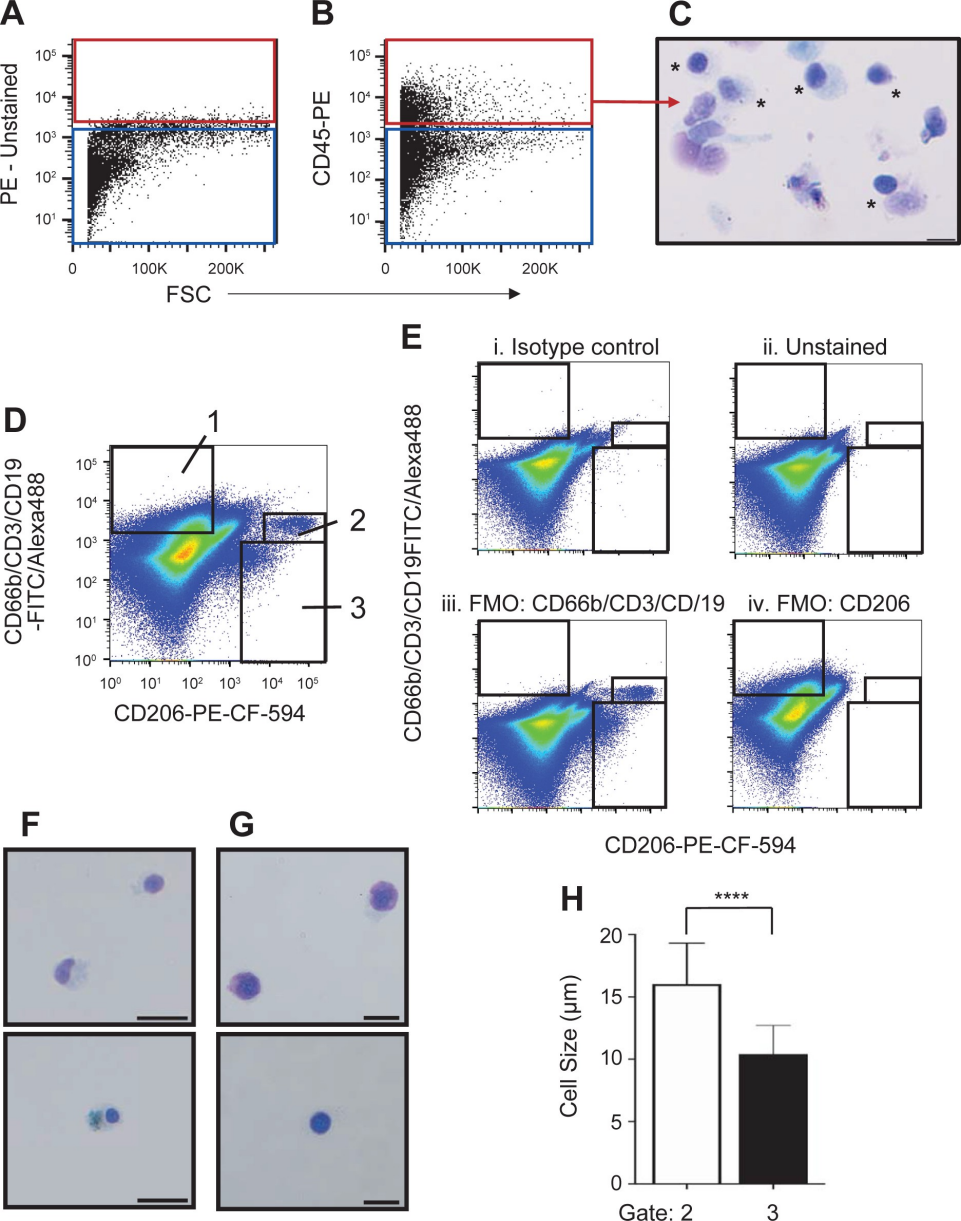
Sputum-derived leukocytes include distinct sub-populations of macrophages. **A-E)** Presented are cells selected through a size exclusion gate and a live cell gate as shown in Fig 1, as well as a doublet discrimination gate (not shown). **A**) Representative light scatter profile of unstained single, live sputum cells defining both the CD45^+^ (red) and CD45^-^ gates (blue) for sorting and further analysis. **B**) Live, single sputum cells stained with the blood panel of antibodies. The red box defines the CD45^+^ sorting population. **C**) Wright-Giemsa-stained cells obtained by sorting the CD45^+^ population, showing various types of leukocytes (*). **D**) Sputum-derived leukocyte profile of FVS510^-^CD45^+^ cells from a different sample stained with the antibodies indicated on the *x-* and *y*-axes. The black boxes indicate the gates used to identify lymphocytes/granulocytes (1), as well as alveolar (2) and interstitial macrophages (3). **E**) Fluorescence minus one (FMO) controls with the same boxes identifying leukocyte subpopulations as defined in **D**. All FMO controls include the viability dye, CD45, and TCPP. **Ei**) FVS510^-^CD45^+^ cells stained with the isotype controls for the antibodies indicated on each axis. **Eii**) Unstained cells. **Eiii**) Sputum cells stained with the leukocyte antibody panel minus the CD66b, CD3, and CD19 antibodies. **Eiv**) Sputum cells stained with the leukocyte antibody panel minus the CD206 antibody. **F, G**) Wright-Giemsa-stained cytospins from the sorted CD45^+^ gate 2 (**F**) and gate 3 (**G**) populations. Scale bars indicate 20 μm in **F** and 10 μm in **G**. Cell types were confirmed by a pathologist. **H**) Cell size measurements of the sorted macrophage population shown in panel **F** (gate 2) and **G** (gate3). For each population at least 100 cells were measured. Presented is the average cell size (+ SD). **** p <0.0001.

Traditionally, the presence of “numerous” macrophages in a sputum smear is indicative of a sample that originates from the lung [52]. We developed a similar quality control measure using the cell surface antigen CD206, which is specific for macrophage populations that reside in lung tissue and are not found in the blood circulation [53]. We stained sputum cells with an antibody directed against CD45 (to identify leukocytes), CD206, and a cocktail of antibodies directed against CD66b, CD3, and CD19 to further separate the macrophages from other hematopoietic cells. FVS510 was used to exclude the dead cells. Fig 3A and 3B show that a proportion of live sputum cells specifically express CD45. Cytospins of sorted CD45^+^ sputum cells confirmed their hematopoietic origin (Fig 3C).

Fig 3D shows a typical profile of CD45^+^ sputum cells labeled with the anti-CD206 antibody and the cocktail of anti-CD66b, anti-CD3 and anti-CD19 antibodies. The isotype control (Fig 3Ei) shows higher background staining than unstained (Fig 3Eii) or fluorescence-minus-one (FMO) controls (Fig 3Eiii and 3Eiv). Since the use of isotype control antibodies comes with its own set of problems [54–56], we used the FMO controls to identify the main subpopulations in sputum. By comparing the FMO control of the CD66b/CD3/CD19 cocktail (Fig 3Eiii) with the stained sample that includes all antibodies (Fig 3D), gate 1 can be set to identify the combined lymphocyte and granulocytes. Similarly, by comparing the FMO control for the CD206 antibody (Fig 3Eiv versus 3D), two populations of CD206-positive cells can be identified (Fig 3D, gates 2 and 3).

After sorting cells from gates 2 and 3, cytological analysis revealed cell populations with a morphology consistent with that of macrophages (Fig 3F and 3G, respectively). However, cells sorted from gate 3 were significantly smaller in size compared to cells sorted from gate 2 (Fig 3H). The sizes we have calculated for the alveolar and interstitial macrophage populations align with size ranges previously reported [57]. Based on the literature, alveolar macrophages are identified as strongly positive for CD206 and autofluorescent in the FITC channel [58] (gate 2 of Fig 3D), while interstitial lung macrophages are smaller in size and lower in CD206 expression [59] (gate 3 of Fig 3D).

The average background staining in the CD206 FMO control was 0.0023% (+/- SD 0.0021%) in both gates combined. A positivity threshold based on 2 standard deviations (SD) above the mean background staining would set it at 0.0065% for both gates combined, or ~ 6 macrophages per 100,000 cells. We were concerned that such a low threshold would not fall within the linear detection range for the PE-CF594 fluorochrome. Therefore, we instead chose an arbitrary threshold of 0.05%, which included alveolar macrophages and interstitial macrophages. This threshold could not by solely based on interstitial macrophages. A 0.05% threshold was well within the linear range of detection of the flow cytometer (S3 Fig) and satisfies the criteria of “numerous macrophages” for an adequate sample as set by the Papanicolaou Society [52].

### Minimum cell number required for reliable flow cytometric analysis

**Fig 4:**
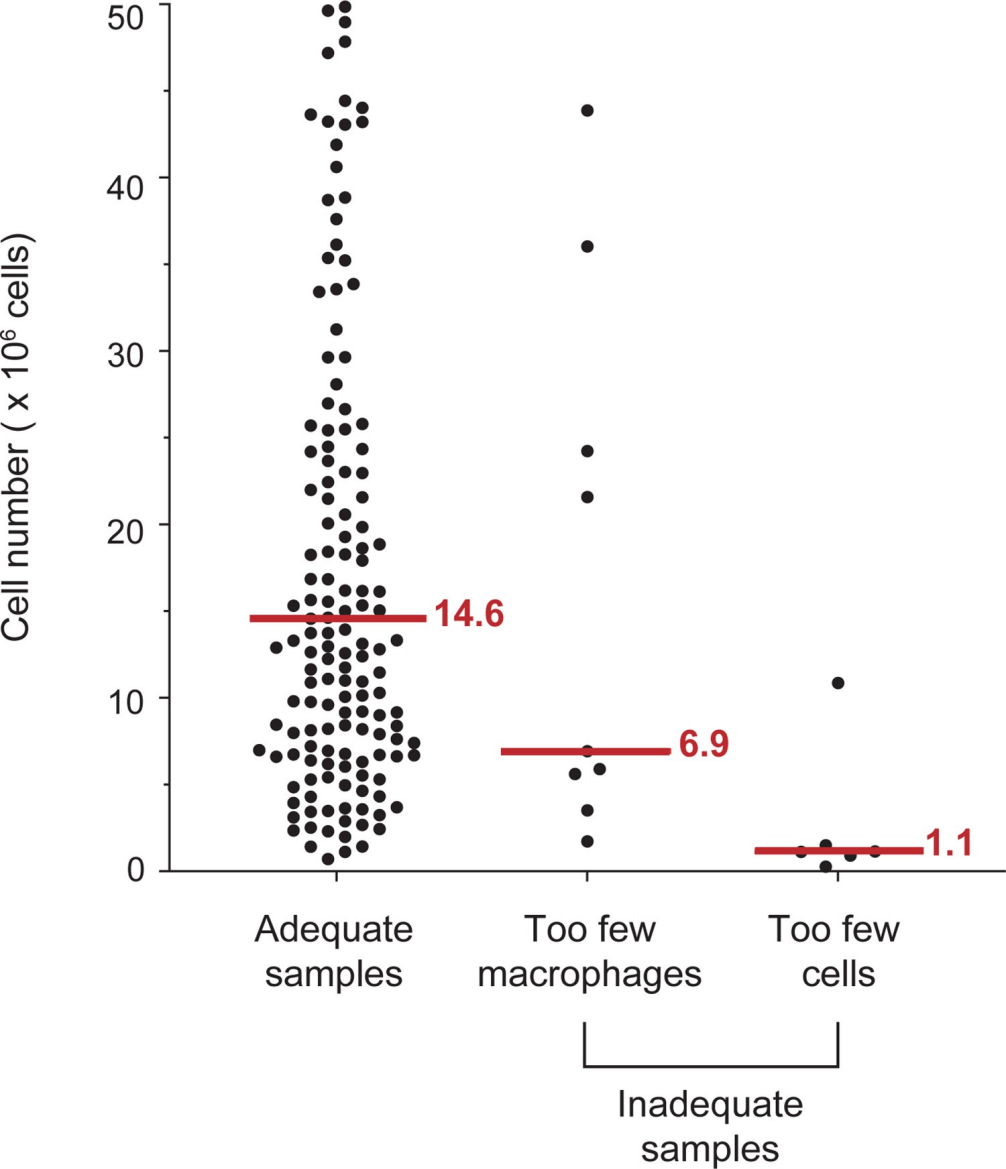
Sample adequacy and criteria for sample exclusion. Shown are the total number of sputum cells (excluding SECs) of individual samples prior to antibody labeling. All adequate samples (n = 164) revealed > 0.05% macrophages (alveolar and interstitial combined). Not depicted are 18 adequate samples, whose sputum cell count exceeded 50 million cells. The inadequate samples (n = 15) either showed no alveolar macrophages or the combined events in the alveolar and interstitial macrophage gates were < 0.05%. A subset of the inadequate samples contained “too few cells” for a reliable profile (< 1000 CD45^+^ events), while the remainder included enough cells, though did not fulfill the QC macrophage criteria to consider them adequate samples. The red lines and numbers indicate the median cell count for each sample group.

One hundred seventy-nine samples were analyzed for macrophage content. Fifteen samples were found to have inadequate macrophage numbers based on the criteria outlined above. However, six of these samples (3.4%) had fewer than 1000 CD45^+^ events for analysis, which are too few cells for an adequate analysis. Five of the six samples had fewer than 1.5 x 10^6^ total sputum cells prior to antibody staining. The remaining nine samples (5.0%) had more than 10,000 CD45^+^ cells (range 11648–463382) and all showed more than 1.7 x 10^6^ sputum cells (Fig 4). Moreover, only four of the 164 adequate samples showed less than 1.5 x 10^6^ cells at the onset of the antibody staining process. Although these samples all included robust macrophage counts, three of the four showed less than 10,000 CD45^+^ cells (range 1327–2908). This data suggests that a sputum sample with less than 1.5 x 10^6^ cells is too small for a reliable diagnostic flow cytometric analysis.

### Identifying differences between cancer and high-risk samples

One hundred sixty-four adequate sputum samples were further analyzed for differences between cancer and high-risk. This set included 32 samples obtained from individuals diagnosed with lung cancer and 132 from high-risk individuals who were cancer-free. The cancer group included 40.6% current smokers and the high-risk group 44.7%. There was no significant difference in pack years smoked between the groups. The average years that the former smokers had quit was also not significantly different. The proportion of females in the cancer group was smaller than in the high-risk group (21.9% versus 54.5%, respectively). The average age of the participants in the cancer group was 69.8 years compared to 64.8 years in the high-risk group (p < 0.0002).

The first phase of the analysis looked at the proportion of CD45^+^ versus CD45^-^ cells and the various subpopulations within each compartment, without the TCPP marker. We found that the proportion of CD45^+^ cells in sputum samples of cancer patients was significantly higher than in sputum from high-risk patients without the disease (49.64% vs 38.95%; p = 0.0099). The different subpopulations of the CD45^+^ compartment as shown in Fig 3D, were recognizable in all samples, however, the relative contribution of each population differed between samples and between groups. By comparing the relative sizes of each CD45^+^ subpopulation between cancer and high-risk samples, we found that cancer samples contained significantly more granulocytes/lymphocytes (gate 1 of Fig 3D; p = 0.0378) and interstitial macrophages (gate 3 of Fig 3D; p = 0.0031).

**Fig 5:**
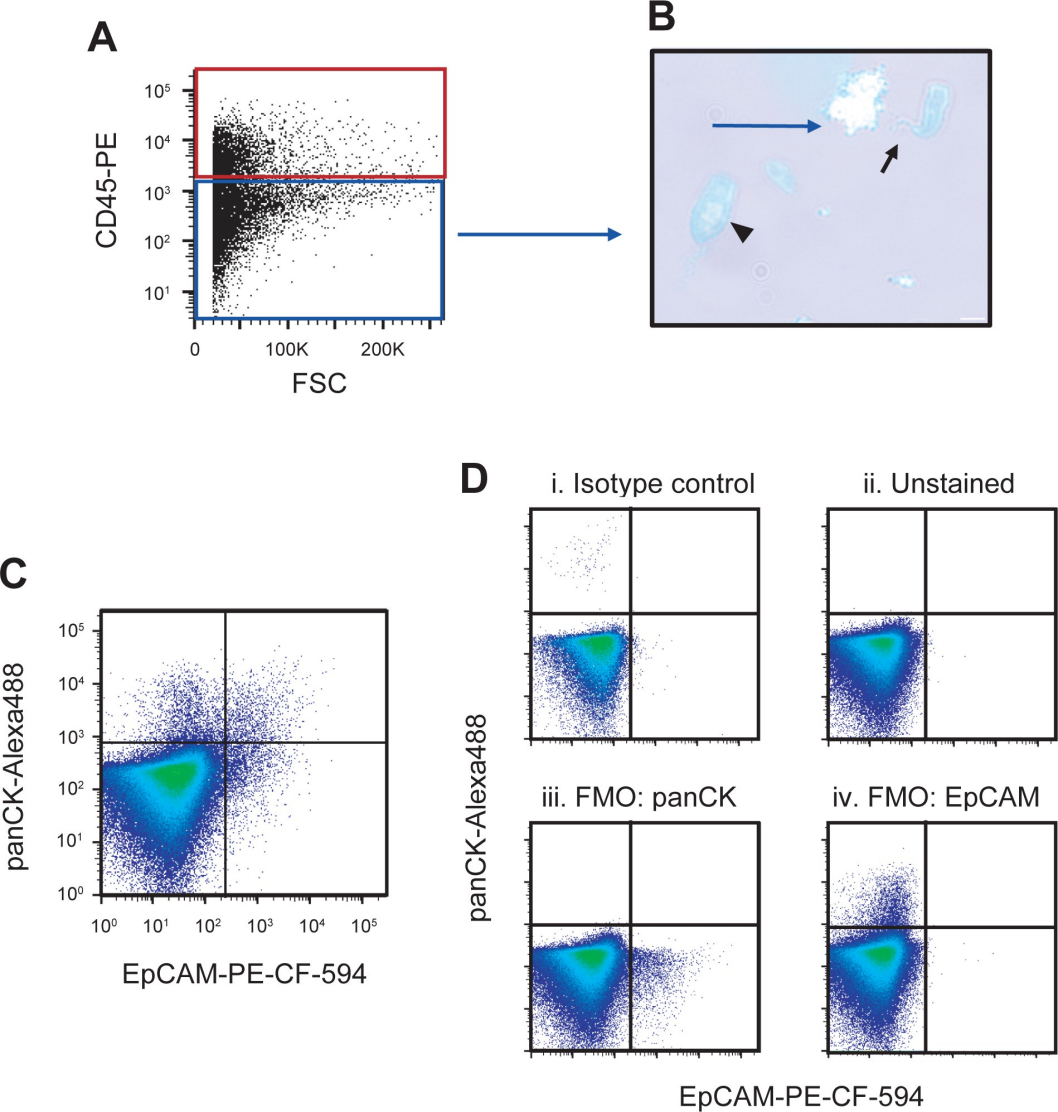
Sputum-derived non-leukocytes identifying epithelial cell populations. All profiles present cells selected through a size exclusion gate and a live cell gate as shown in Fig 1, as well as a doublet discrimination gate (not shown). **A**) The same cell suspension used to sort CD45^+^ cells as presented in Fig 3, was used to sort the CD45^-^ fraction (blue gate). **B**) Wright-Giemsa-stained CD45^-^ cells. The arrow indicates a ciliated epithelial cell and arrowhead indicates a goblet cell. All images were confirmed by a pathologist. Scale bars indicate 20 μm. **C**) Live, single CD45^-^ sputum cells, from a different sample, stained with the epithelial antibody panel. **D**) Fluorescence minus one (FMO) controls for the profile presented in **C**. FMO controls include viability dye, CD45, and TCPP. **Di**) Sputum-derived epithelial profile of FVS510^-^CD45^-^ cells stained with the isotype controls for the antibodies used in **C**. **Dii**) Unstained sputum cells. **Diii**) FMO control FVS510^-^CD45^-^ cells stained with EpCAM but without the panCK antibody. **Div**) FMO control FVS510^-^CD45^-^ cells stained with panCK but without the EpCAM antibody.

The CD45^-^ compartment (Fig 5A, blue box) includes cells of epithelial origin, which was confirmed by the presence of goblet and ciliated epithelial cells when CD45^-^ cells were sorted and their morphology visualized on cytospins (Fig 5B). Using antibodies directed against EpCAM and cytokeratins [60] allowed us to further delineate the CD45^-^ cells by flow cytometry (Fig 5C). The FMO controls show the low background of the respective antibodies used (Fig 5D). The relative contribution of the various CD45^-^ subpopulations varied from sample to sample and no significant differences were observed between the cancer and high-risk groups.

**Fig 6:**
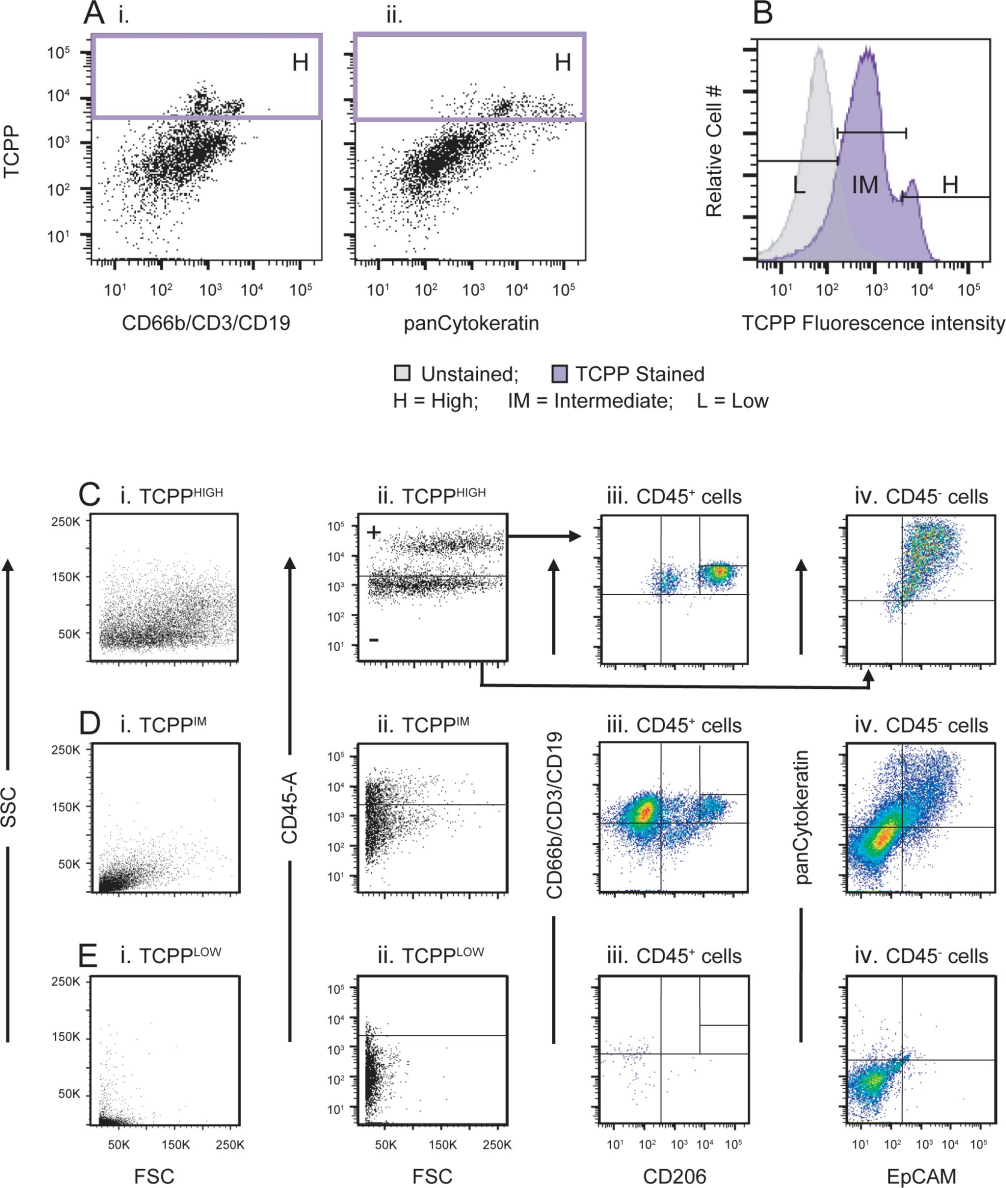
Sputum cells highly stained with TCPP represent unique subpopulations. **A, B**) Defining sputum cell populations with different TCPP fluorescence intensities (FI). **A**). The dot plot displaying TCPP versus FITC/Alexa488 fluorescence (i.e., CD66b/CD3/CD19 in the leukocyte tube (Ai) and panCK in the non-leukocyte tube (Aii)) is used to define the TCPP^HIGH^ cut-off. The TCPP versus PE-CF594 fluorescence plot (not shown) can also be used for this purpose but the cells with the highest FI for TCPP are easier to identify in the former. **B**) Representative histogram of the TCPP FI. The TCPP^HIGH^ cut-off is taken from the gate shown in panel **A**. The TCPP^LOW^ population is defined at the intersect when unstained sputum is overlaid with the TCPP-stained sample. The population with intermediate TCPP staining, TCPP^IM^, is defined as the population between the TCPP^HIGH^ and the TCPP^LOW^ populations. Each of the three TCPP populations identified in **B** are further analyzed in **C–E**. Row **C** represents the TCPP^HIGH^ subset analysis, row **D** the TCPP^IM^ analysis and row **E** the TCPP^LOW^ analysis. The first profile of each row (**column i**) shows the light scatter profile of the respective TCPP subpopulation. The second profile of each row (**column ii**) shows the distribution of CD45 staining of the cells in the respective TCPP subpopulation. The CD45^+^ fraction (“+”) of each TCPP subpopulation is further analyzed in **column iii**, which shows the distribution of CD66b/CD3/CD19 staining versus CD206. The CD45^-^ ("-") fraction of each TCPP subpopulation is represented in **column iv**, which shows the panCytokeratin versus EpCAM staining.

The second phase of the analysis looked at TCPP fluorescence. Single, live cells were separated into three subsets of cells based on TCPP staining intensity: TCPP^HIGH^, TCPP^INTERMEDIATE(IM)^ and TCPP^LOW^ cells (Fig 6A and 6B). The relative ratios of these subsets of cells did not differ between the high-risk and cancer groups. Each of these three subsets were then further interrogated for their content of CD45^+^ leukocyte populations (columns ii and iii) and CD45^-^ epithelial cell populations (columns ii and iv). The analysis of TCPP^HIGH^ cells is depicted in (Fig 6Ci–6Civ), the analysis of TCPP^IM^ cells in (Fig 6Di–6Div), and the analysis of TCPP^LOW^ cells in (Fig 6Ei–6Eiv). TCPP^HIGH^ cells display a broad light scatter profile (Fig 6Ci). The CD45^+^ compartment of TCPP^HIGH^ cells (Fig 6Cii) are enriched for alveolar macrophages (CD45^+^;CD206^++^ cells; Fig 6Ciii) while the CD45^-^ compartment is enriched for EpCAM^+^;panCK^+^ double positive cells (Fig 6Civ). The TCPP^IM^ cells represent most of the sputum cells and thus the profile of this subpopulation resembles that of the entire sample. The TCPP^LOW^ cells displayed relatively low light scatter properties compared to TCPP^HIGH^ cells (Fig 6Ei) and they are mostly CD45^-^ (Fig 6Eii) without expression of the epithelial markers EpCAM or panCK (Fig 6Eiv).

**Fig 7:**
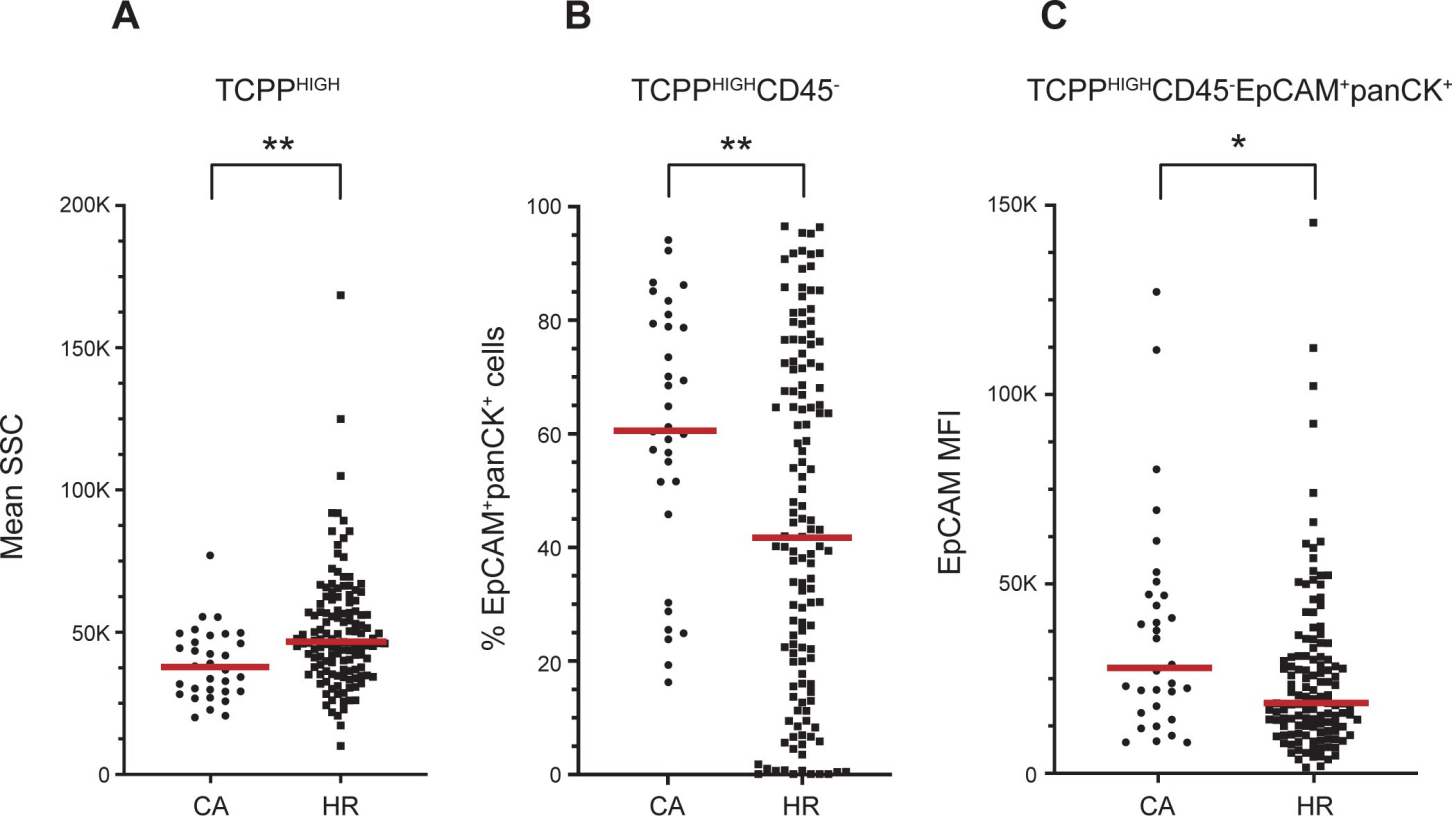
Differences in sputum cell characteristics between cancer and high-risk sputum samples. Depicted are the significant differences between cancer (CA; n = 32) and high-risk samples (HR; n = 132) resulting from the analysis described in Fig 5. Each dot (CA) and square (HR) represent one sample. **A**) the TCPP^HIGH^ population in cancer samples displays a smaller SSC than the TCPP^HIGH^ population in high-risk samples (** p <0.01). **B**) In cancer samples, the proportion of EpCAM^+^panCK^+^ cells in the CD45^-^ fraction of the TCPP^HIGH^ subpopulation is larger than in the corresponding CD45^-^ fraction in high-risk samples (** p < 0.01). **C**) The mean fluorescence intensity (MFI) of EpCAM in TCPP^HIGH^ CD45^-^EpCAM^+^panCK^+^ cells is higher in cancer samples than in the corresponding cellular subset of high-risk samples (* p < 0.05). The red lines indicate the median values for each sample group.

The unique properties of the TCPP^HIGH^ population showed several significant differences between the high-risk and cancer groups. First, the TCPP^HIGH^ cells from samples of the cancer group showed lower side scatter values than those from the high-risk group (Fig 7A). Second, the CD45^-^ compartment of the TCPP^HIGH^ population contained a higher percentage of EpCAM^+^panCK^+^ cells (Fig 7B). Additionally, this double positive population from samples in the cancer group expressed higher levels of EpCAM, though not panCK, compared to the cells of the same quadrant that belonged to samples from the high-risk group (Fig 7C).

Upon further analysis of the cancer group, we observed significantly higher EpCAM mean fluorescence intensity in early-stage cancer samples (Stage I/II) compared to later stage cancer samples (Stage III/IV), (p = 0.047). We did not find any significant differences based on cancer type (squamous cell carcinoma versus adenocarcinoma) nor any differences based on smoking history (current versus former smokers). Interestingly, when we separated high-risk smokers based on smoking history, the profiles of current high-risk smokers showed the presence of significantly more TCPP^HIGH^;EpCAM^+^;panCK^+^ cells (p = 0.0008) as well as macrophage populations, both alveolar (p = < 0.0001) and interstitial (p = 0.0141) compared to former smokers.

## Discussion

Current methodologies used for sputum analysis pose challenges that have limited their clinical use. Sputum cytology suffers from low sensitivity due to the high skill required for identifying subtle nuclear changes. The need to screen numerous slides makes it time consuming, which also hampers its clinical use [61–63]. Imaging and molecular techniques can assess genetic changes in sputum-derived cells but screening methods based on nuclear ploidy or in situ hybridization to detect genetic abnormalities use only several hundred cells per sputum sample [61, 62, 64], and microchip analysis of enriched epithelial cells derived from sputum-analyzed genetic aberrations screen only 2000 cells per slide [63]. The exclusion of the majority of sputum cells from analysis may hide important disease parameters, leading to lower sensitivity than is clinically helpful [64]. The limitations of these various technologies should not be conflated with the highly useful nature of sputum as a biological fluid, which can provide an important cellular snapshot of the lung environment.

The flow cytometric platform is well suited to analyze exfoliated cells isolated from sputum for identifying tumor-related changes in leukocyte and non-leukocyte populations that would otherwise go undetected by conventional cytological methods [65]. The ability to detect and analyze cells based on their physical characteristics (i.e., size and granularity) and cell surface molecules is rivaled by microscopy techniques [18, 66]. Unlike microscopy or cytology, however, flow cytometry can analyze large numbers of cells in a short time.

The variability in autofluorescence and non-specific binding properties of cell populations within and between sputum samples prohibit the use of commercially available biological controls, often used in immunophenotyping of highly characterized hematopoietic populations. For this reason, an internal FMO control has been used to establish a positivity threshold for the macrophage gates [67]. The ability to identify alveolar macrophages as a distinct leukocyte subpopulation allowed us to include a built-in flow cytometry quality control parameter for determining the lung origin of each sputum sample. We therefore did not have to rely on cytology-based sample quality confirmation.

The lungs are continuously exposed to pathogens and noxious particulates and alveolar macrophages are the predominant, primary innate defense for maintaining a healthy lung environment [68]. Alveolar macrophages have been characterized as a distinct CD45^+^ population with high CD206 expression (CD206^++^) and a moderate-to-high signal on the granulocyte/lymphocyte axis due to their autofluorescence [58, 65]. Additionally, our results confirmed previous observations where the light scatter profile of alveolar macrophages overlapped with that of contaminating SECs [69, 70], highlighting the need of sequestering SECs from further analysis.

CD206-intermediate-positive cells (CD206^+^) are also macrophages though they are smaller than the alveolar CD206^++^ macrophages and display minimal FITC auto-fluorescence, indicating this macrophage population likely represents interstitial macrophages [53, 65, 71, 72]. Although interstitial macrophages (as opposed to alveolar macrophages) are normally not in contact with the airway lumen, the pro-inflammatory environment caused by chronic smoking is ideal for the permeation of interstitial macrophages into the airway [68]. Their presence in sputum obtained from heavy smokers is therefore not unexpected. This is further substantiated by our finding that current high-risk smoker have significantly more macrophages in their sputum than former high-risk smokers.

The minimum number of sputum-derived cells required to give an adequate profile so the macrophage presence could be determined was approximately 1.5 million cells. The cutoff of five macrophages per 10,000 cells (0.05%) for determining sample adequacy was well within the detection range of the flow cytometer. Interstitial macrophages were included in the 0.05% macrophage cutoff for sample adequacy because the presence of both alveolar and interstitial macrophages are lung tissue-specific cell populations. The presence of interstitial macrophages without the presence of alveolar macrophages (a rare occurrence) is difficult to interpret biologically, therefore samples without any alveolar macrophages were deemed inadequate. These parameters for limits of detection are a rough indication and still need to be validated according to the official guidelines for flow cytometric assay development [73].

A comparative, multi-parameter analysis of sputum samples from persons with confirmed lung cancer versus those from persons at high-risk of developing the disease revealed significant differences between the two groups. Cancer samples contained significantly more CD45^+^ cells than high-risk samples, specifically more granulocytes/lymphocytes and interstitial macrophages. The addition of the porphyrin TCPP to our staining protocol allowed us to identify several significant differences in the most brightly stained subset (TCPP^HIGH^) between the cancer and high-risk groups. TCPP^HIGH^ cells from the cancer group, irrespective of their CD45 lineage, displayed lower side scatter properties than TCPP^HIGH^ cells from the high-risk group, suggesting a decrease in cytoplasm content, organelle degranularization, and vacuolization that has been documented with malignancy [74]. Analysis of the non-leukocyte (CD45^-^) subpopulation of TCPP^HIGH^ cells revealed that the cancer group contained a larger percentage of cells stained with the epithelial markers panCK and EpCAM. This difference with the high-risk group is caused mainly by the fact that former high-risk smokers have significantly fewer of these cells in their sputum compared to current smokers. The epithelial cell subpopulation from the cancer group expressed also higher levels of EpCAM, though equal levels of panCK. This was most noticeable in the Stage I/II subgroup.

Detection of epithelial-derived cancers and circulating tumor cells historically has relied on the detection of both EpCAM and cytokeratin expression [75–77]. Our flow cytometry-based analysis that identifies increased expression of EpCAM in Stage I/II cancer-confirmed samples, as well as samples from high-risk participants who continue to smoke, suggests that EpCAM expression may be of specific importance in early lung cancer detection.

We did not detect any significant differences in TCPP fluorescence in the cancer versus high-risk macrophage populations, though the literature suggests an increased uptake in porphyrins by tumor-associated macrophages [78, 79]. This lack of macrophage significance may be due to the protocol we developed, where TCPP staining occurred post-fixation and not with live sputum-derived cells.

The flow cytometry approach described in this study may be modified to target specific immune cell populations, or other cell types such as endothelial or apoptotic cells, that may potentially improve its ability to distinguish cancer samples from high-risk sputum samples. In addition, advances in automation and machine learning offer the potential to discover novel cell populations or biomarkers that might have gone unnoticed by manual analysis of flow cytometry data conducted in our current study [80, 81]. Automated analysis furthermore eliminates two debilitating constraints of manual analysis: bias and excessive time for analysis. Applying automated analysis is therefore necessary for advancing to a clinically relevant diagnostic test for the detection of lung cancer.

In summary, the present study shows that flow cytometric analysis represents a platform capable of identifying reproducible cellular populations from whole sputum samples. Additionally, our built-in quality control for the detection of lung-specific macrophages ensures the adequacy of samples analyzed while the elimination of contaminating dead cells (including SECs), debris, and doublets ensures high-quality analysis. Our emphasis was on lung cancer, but this flow cytometric platform can be amenable to other pulmonary diseases, including COVID-19. Irrespective of the intended use, adaptation of sputum flow cytometry for high-throughput clinical diagnosis still requires automation and other standardization procedures.

## Supporting information

S1 Fig Filtration of three-day induced sputum samples does not eliminate squamous epithelial cells (SECs). (PDF)

S2 Fig Light scatter profiles of cell types commonly found in sputum. (PDF)

S3 Fig Range of detection for the PE-CF594 fluorochrome. (PDF)

S1 Table Reagents used for sputum staining and flow cytometric analysis. (PDF)

S2 Table Size estimation for cells that can be found in the lung. (PDF)
