# Supplementary material for: Sputum analysis by flow cytometry; an effective platform to analyze the lung environment
Source: PLoS One. 2022 Aug 17;17(8):e0272069. doi: 10.1371/journal.pone.0272069 (PMC9385012; doi:10.1371/journal.pone.0272069)
Supplement: S1 Fig — (PDF) [file pone.0272069.s001.pdf]

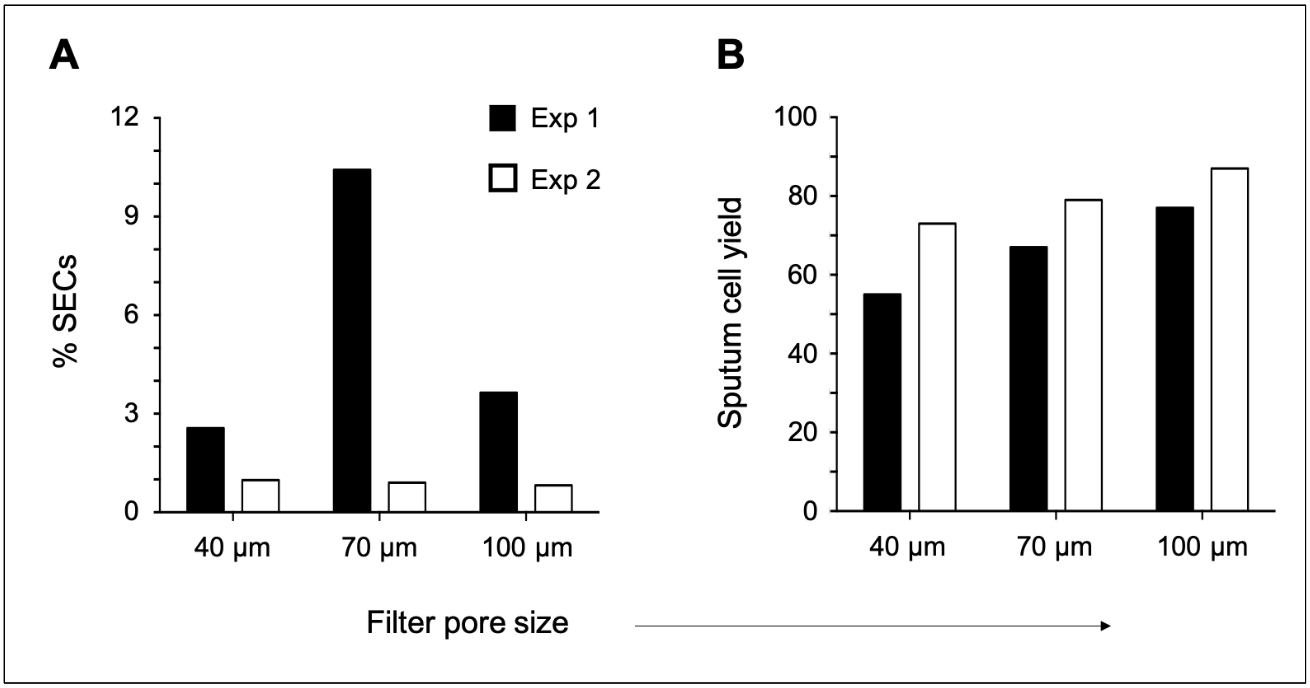

S1 Figure. Filtration of three-day induced sputum samples does not eliminate squamous epithelial cells (SECs). A) Dissociated sputum from two individual experiments was filtered through strainers of various pore size and the percent of the SECs was calculated from the total sputum-derived cell count. Cell suspensions ran smoothly through the 100 µm and 70 µm filters while the 40 µm filter easily clogged. The overall proportion of SECs in the samples after filtration were similar, irrespective of filter pore size. **B**) The concentration of dissociated sputum cells from two individual experiments was determined prior to filtration and again post-filtration. Presented is the sputum cell yield after filtration as a percentage of starting cell number. These results show that none of the filters selectively trap SECs, but instead trapped a proportion of the sample non-specifically. It was decided to use the 100 µm filter in our processing protocol so that the larger debris could be captured while minimizing cell loss.
