# Supplementary material for: Sputum analysis by flow cytometry; an effective platform to analyze the lung environment
Source: PLoS One. 2022 Aug 17;17(8):e0272069. doi: 10.1371/journal.pone.0272069 (PMC9385012; doi:10.1371/journal.pone.0272069)
Supplement: S2 Fig — (PDF) [file pone.0272069.s002.pdf]

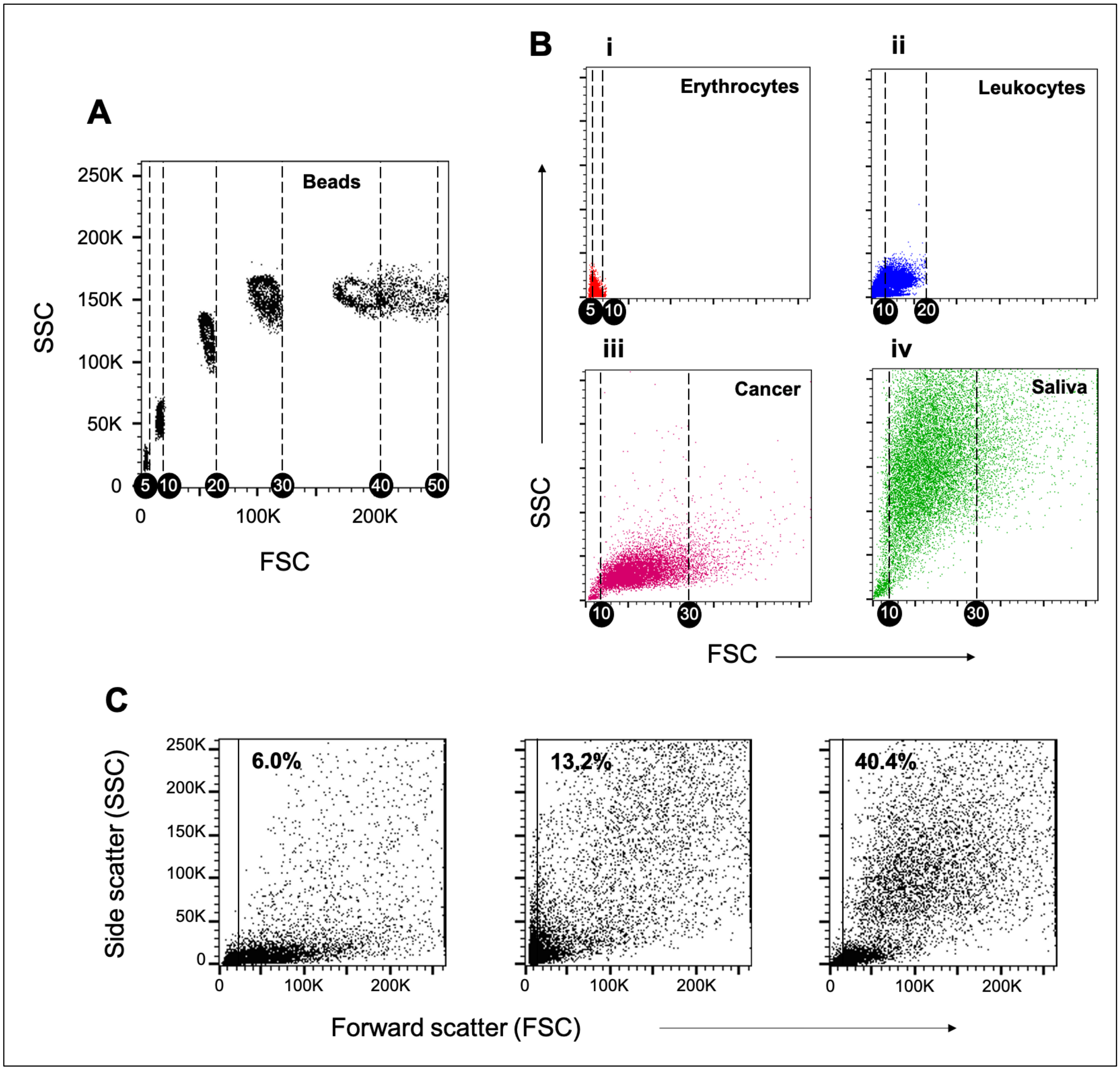

S2 Figure. Light scatter profiles of cell types commonly found in sputum. (**A**) Light scatter profiles of NIST bead particles of known diameter, with the sizes (µm) indicated on the x- axis as black circles. These same black circles indicating the size diameter based on the NIST bead particle scatter profiles are displayed on the axes of profiles Bi through Biv. (**B**) Size ranges for each of the indicated cell types: i Erythrocytes; ii Leukocytes; iii HCC15 cancer cells; iv Saliva including mostly squamous epithelial cells (SECs). The profiles of the erythrocytes, leukocytes, and cancer cells (**Bi – Biii**) show that their respective FSC ranges

correspond to that of the expected bead size range (See S2 Table for expected cell sizes). Since these cell populations constitute our cells of interest, we used 5, 20 and 30 μm NIST beads as controls (with consistent laser voltages) to standardize the first step of the visualization of sputum cells in our analysis. Based on the literature (S2 Table), the FSC of saliva cells was expected to be larger than the 50 μm beads. Instead, we found their FSC was similar to that of leukocytes and cancer cells (**Biv**). Although the SSC properties of saliva cells are for the most part much larger than that of blood and cancer cells, there was a fraction of saliva cells that overlapped with blood and cancer cells. Therefore, neither FSC nor SSC parameters could be used to eliminate SECs from the analysis. (**C**) Scatter profiles of three independent dissociated sputum samples with various amounts of SEC contamination (noted for each sputum sample at the top left of each scatter profile). The far-right profile shows a noticeable SECs population with a wide range in FSC and SSC parameters (compare to **Biv**). Based on this data it was decided that neither FSC nor SSC parameters could be used to exclude SEC from the sputum analysis.
