# Supplementary material for: Sputum analysis by flow cytometry; an effective platform to analyze the lung environment
Source: PLoS One. 2022 Aug 17;17(8):e0272069. doi: 10.1371/journal.pone.0272069 (PMC9385012; doi:10.1371/journal.pone.0272069)
Supplement: S3 Fig — (PDF) [file pone.0272069.s003.pdf]

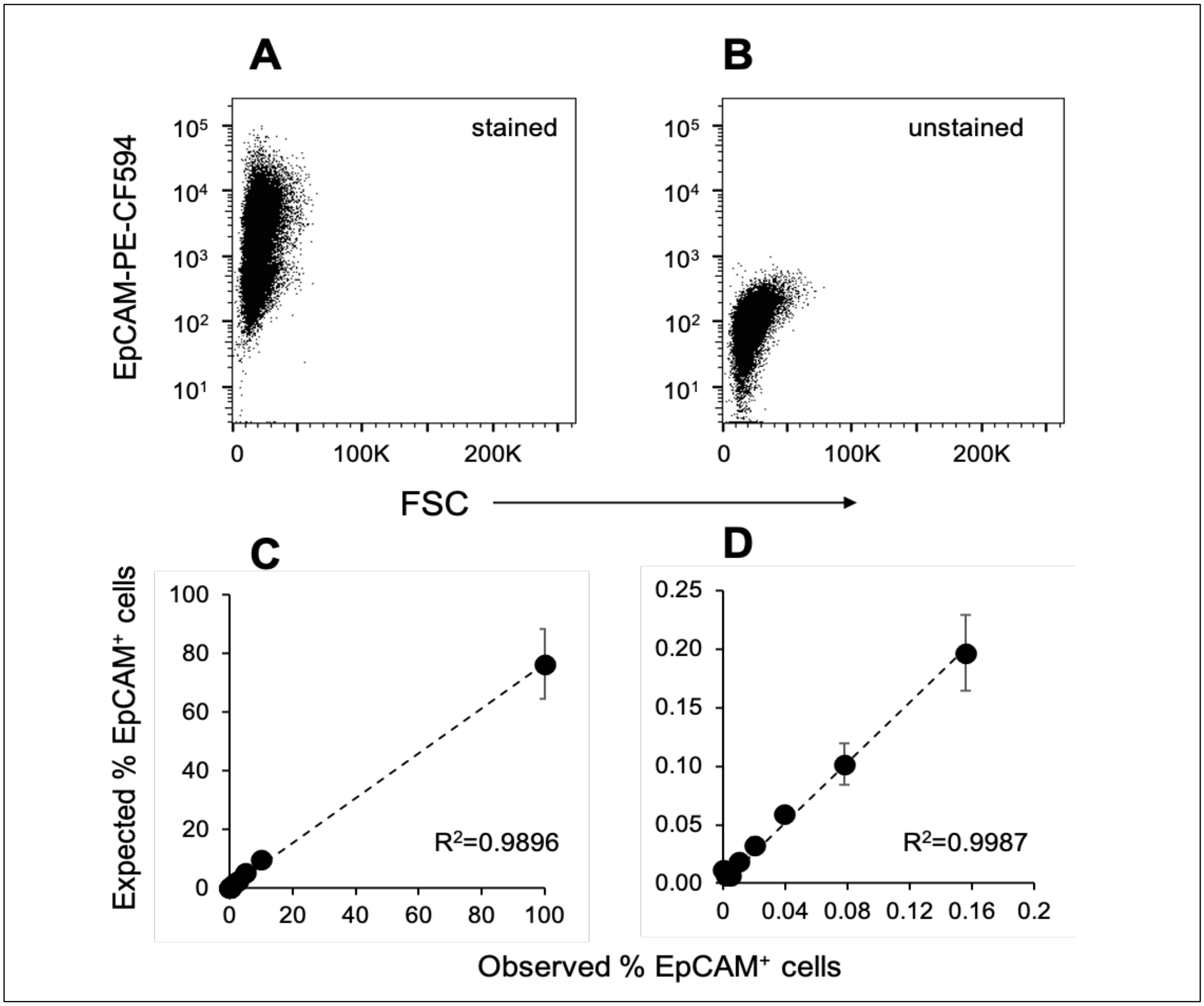

S3 Figure. Range of detection for the PE-CF594 fluorochrome. (**A-B**) We performed cell dilution experiments to establish the linear range of detecting events with a PE-CF594 fluorochrome for the flow cytometer we used for all our experiments. Since there is no cell line available that stains 100% with the CD206 antibody, we used a substitute cell line and antibody: A549 human lung cancer cells and EpCAM-PE-CF594. (**A**) All A549 lung cancer cells stain positive for EpCAM compared to unstained cells (**B**). The FVS510 viability dye was included to eliminate dead cells. (**C-D**) Serial dilutions of A549 cells stained with EpCAM-PE-CF594 and FVS510 were made with A549 cells that were only labeled with FVS510. Each dilution was then analyzed by flow cytometry and the percentage of live, single EpCAM-PE-CF594-positive cells determined. The percent expected and observed

EpCAM-PE-CF594-positive cells were plotted against each other. Linear regression analysis of all data points is shown in (**C**) and a subset of the data points (those representing the most diluted samples is shown in (**D**). (n = 3).

The unstained control showed on average 0.012% background events in the PE-CF594 channel. Regression analysis of the data revealed a very good linear relationship between the expected and observed proportion of EpCAM-positive cells, even when the percent labeled cells was very low (**D**). These results indicate that the flow cytometer reliably detects very small number of cells labeled with a PE-CF594 fluorochrome.
