# Supplementary material for: Sputum analysis by flow cytometry; an effective platform to analyze the lung environment
Source: PLoS One. 2022 Aug 17;17(8):e0272069. doi: 10.1371/journal.pone.0272069 (PMC9385012; doi:10.1371/journal.pone.0272069)
Supplement: S1 Table — (PDF) [file pone.0272069.s004.pdf]

S1 Table. Reagents used for sputum staining and flow cytometric analysis

Antibody/stain	Vendor	Final concentration	Clone	Catalog Number
Anti-hCD45 – PE	BioLegend (San Diego, CA)	1 μg/mL	HI30	304039
Anti-hCD66b – FITC	GeneTex (Irvine, CA)	3 μg/mL	80H3	GTX75907
Anti-hCD3 – Alexa488	BioLegend (San Diego, CA)	2 μg/mL	UCHT1	300415
Anti-hCD19 – Alexa488	BioLegend (San Diego, CA)	2 μg/mL	HIB19	302219
Anti-hCD206 – PE-CF594	BD Biosciences (San Jose, CA)	3 μg/mL	19.2	564063
Anti-hEpCAM – PE-CF594	BD Biosciences (San Jose, CA)	1 μg/mL	EBA-1	565399
Anti-hpanCK – Alexa488	BioLegend (San Diego, CA)	4 μg/mL	C-11	628608
FVS510	BD Biosciences (San Jose, CA)	6 – 10X	N/A	564406
TCPP	Frontier Scientific (Logan, UT)	10 μg/mL	N/A	T790
Paraformaldehyde	Polysciences (Warrington, PA)	1%	N/A	25037
Mouse IgG1κ – Alexa488	BioLegend (San Diego, CA)	4 μg/mL	MOPC-21	400129
Mouse IgG1κ – FITC	BD Biosciences (San Jose, CA)	3 μg/mL	MOPC-21	555748
Mouse IgG1κ – PE-CF594	BD Biosciences (San Jose, CA)	3 μg/mL	X-40	562292
C&ST beads***	BD Biosciences (San Jose, CA)	N/A	N/A	655051
5 μm Microbead NIST Traceable Particle Size Standards*	Polysciences (Warrington, PA)	4 x 10^5/mL	N/A	64080
20 μm Megabead NIST Traceable Particle Size Standards	Polysciences (Warrington, PA)	2 x 10^5/mL	N/A	64160
30 μm Megabead NIST Traceable Particle Size Standards	Thermo Fisher Scientific (Waltham, MA)	2 x 10^5/mL	N/A	4230A
Anti-mCompBead Plus**	BD Biosciences (San Jose, CA)	N/A	N/A	560497

h = human; panCK = pan-cytokeratin; m = mouse; all antibodies are monoclonal

* For research purposes 10 μm, 40 μm, and 50 μm Megabead NIST Particle Size Standards were also used (S2 Fig).

** Concentrations not determined; used per manufacturer's protocol

All reagents were titrated using sputum from persons at high-risk of developing lung cancer.
