# Supplementary material for: Sputum analysis by flow cytometry; an effective platform to analyze the lung environment
Source: PLoS One. 2022 Aug 17;17(8):e0272069. doi: 10.1371/journal.pone.0272069 (PMC9385012; doi:10.1371/journal.pone.0272069)
Supplement: S2 Table — (PDF) [file pone.0272069.s005.pdf]

S2 Table. Size estimation for cells that can be found in the lung.

Cell Type	Diameter (μm)	Reference
**Hematopoietic cells**		
Erythrocytes	6-8	Wheater *et al*.
Granulocytes	9-12	Wheater *et al*.
Monocytes	14-17	Wheater *et al*.
Lymphocytes	7-8	Wheater *et al*.
**Other**		
Alveolar macrophages	21	Krombach *et al.*
Type I alveolar epithelial cell (lung cells)	Up to 50	Kini
Type II alveolar epithelial cells (lung cells)	9-15	Kini
Lung cancer cells (HCC15 cells)	20-30	Fillmore *et al.*
Squamous epithelial cells (cheek cells)	65	Paszkiewicz *et al.*

References:

Wheater PR, Burkitt HG, Daniels VG. Functional histology. 1st edition. Norwich, England: Jarrold & Sons Ltd; 1979.

Krombach F, Münzing S, Allmeling AM, Gerlach JT, Behr J, Dörger M. Cell size of alveolar macrophages: an interspecies comparison. Environ. Health Perspect. 1997;105(Suppl 5):1261–1263. doi: 10.1289/ehp.97105s51261

Kini SR. Color atlas of pulmonary cytopathology. 1st edition. New York, USA: Springer-Verlag New York, Inc.; 2002.

Fillmore CM, Xu C, Desai PT, Berry JM, Rowbotham SP, Lin YJ, et al. EZH2 inhibition sensitizes BRG1 and EGFR mutant lung tumors to TopoII inhibitors. Nature. 2015;520(7546):239–242. doi: 10.1038/nature14122

Paszkiewicz GM, Timm EA, Mahoney MC, Wallace PK, Sullivan Nasa MS, Tammela TL, et al. Increased human buccal cell autofluorescence is a candidate biomarker of tobacco smoking. Cancer Epidemiol. Biomarkers Prev. 2008;17(1):239–244. doi:10.1158/1055-9965.EPI-07-0162
